# Optimal GRID-HAM-D7 cut-off scores for defining remission in older Thai adults with depression

**DOI:** 10.3389/fpsyt.2026.1678542

**Published:** 2026-01-30

**Authors:** Nahathai Wongpakaran, Rewadee Jenraumjit, Peerasak Lerttrakarnnon, Thanitha Sirirak, Nopporn Tantirangsee, Rob van Reekum, Tinakon Wongpakaran

**Affiliations:** 1 Faculty of Medicine, Chiang Mai University, Chiang Mai, Thailand; 2 Department of Pharmaceutical Care, Faculty of Pharmacy, Chiang Mai University, Chiang Mai, Thailand; 3 Faculty of Medicine, Prince of Songkla University, Songkhla, Thailand; 4 Songkhla Rajanagarindra Psychiatric Hospital, Songkhla, Thailand; 5 Department of Psychiatry, Faculty of Medicine, University of Toronto, Toronto, ON, Canada

**Keywords:** diagnostic threshold, elderly population, GRID-HAM-D7, late-life depression, remission

## Abstract

**Objectives:**

The 7-item Hamilton Depression Rating Scale (HAM-D7) is commonly used to assess depression severity and remission; however, its standard cut-off scores may not be optimal for elderly populations. This study aimed to establish GRID-HAM-D7 remission thresholds among elderly Thai patients diagnosed with depressive disorders, including both any depressive disorder and major depressive disorder (MDD).

**Methods:**

A total of 803 elderly participants were recruited from four tertiary care hospitals across Thailand as part of a larger psychiatric study. Diagnoses were determined using the Mini International Neuropsychiatric Interview, and depression severity was assessed via the GRID-HAM-D7. Statistical analyses, including sensitivity, specificity, predictive values, and Receiver Operating Characteristic curves, were performed to determine optimal remission cut-off scores.

**Results:**

For any depressive disorder, a GRID-HAM-D7 score of ≤ 4 yielded sensitivities of 88.86% and specificity of 77.66%. In major depressive disorder, the optimal threshold was ≤ 6, resulting in 91.68% sensitivity and 79.73% specificity. Both values surpassed the diagnostic accuracy of conventional lower thresholds. These results suggest that higher GRID-HAM-D7 remission cut-offs better reflect depressive symptomatology in older adults.

**Conclusions:**

The study underscores the necessity of tailoring standardized assessment tools for specific populations to enhance clinical management and decision-making in geriatric psychiatry.

## Introduction

Major depressive disorder (MDD) is a profound global health concern, especially among older adults. Depression in this population is common, frequently under-recognized, and often undertreated. With a prevalence rate of 31.74% among the global elderly population (1), the need for regular screening and effective interventions is clear. This high rate highlights the importance of proactive strategies for detection and management in older adults. Recognizing these challenges, clinicians require reliable and efficient tools for accurate assessment and monitoring in geriatric psychiatry.

In many countries, the numbers are rising due to demographic shifts resulting in rapidly aging populations. Among the elderly, depressive presentations are often complex and may include chronic low-grade symptoms that precede or coexist with major depressive episodes, a condition known as *double depression*. Double depression refers to the co-occurrence of persistent depressive disorder (PDD), former dysthymia, and major depressive disorder (MDD). It is associated with greater symptom severity, longer duration, and poorer treatment outcomes (2). Beyond the immediate emotional distress, depression in the elderly is associated with a significant increase in disability, poorer outcomes in physical illness, and considerably diminished quality of life (3). Furthermore, untreated or poorly managed depression in this population is linked to increased health care utilization and mortality, including suicide (4, 5).

Clinicians often face substantial challenges in the diagnosis and management of late-life depression (6). Presentation of depressive symptoms in older adults can be atypical, with core symptoms such as persistent sadness or hopelessness sometimes masked by physical complaints, cognitive impairment, anhedonia (loss of interest), sleep disturbances, and prominent fatigue. Some elderly patients may have difficulty articulating mood-related distress or may attribute symptoms to aging or medical illnesses. Thus, standard diagnostic systems such as DSM-5 and ICD-10, while widely used and generally effective, may not fully capture the unique symptom presentation in older adults (7).

In treating depression among the elderly, ongoing monitoring of symptoms is vital for evaluating treatment response, adjusting therapeutic interventions, and supporting shared decision-making between clinicians and patients. The use of standardized rating scales is now routine in both clinical trials and real-world practice, allowing for the systematic tracking of symptom severity and remission status over time. Among these, clinician-rated scales are particularly valuable for ensuring objective assessment, especially in populations with cognitive impairment or poor insight (8–10).

The Hamilton Depression Rating Scale (HAMD), initially developed in 1960, is one of the most widely used clinician-administered depression rating scales globally (11). The 17-item version (HAM-D17) was designed to evaluate a range of depressive symptoms and has become the gold standard for depression severity assessment and for assessing treatment efficacy in both research and clinical practice. Other expanded versions, such as HAM-D21 and HAM-D24, have been used for similar purposes.

Given the diversity of patient populations and care environments, shorter HAMD versions have been introduced to address practical challenges such as patient fatigue, cognitive limitations, and clinical time constraints. Notably, while Goldman and colleagues recently studied a 6-item form for rapid assessment (12), our study specifically utilizes the 7-item HAM-D7 validated by McIntyre and colleagues (13). This version targets a core set of depressive symptoms and is especially suited to older adults who may benefit from briefer evaluations without sacrificing diagnostic accuracy.

The HAM-D7 includes the following items: (1) Depressed mood, (2) Feelings of guilt, (3) Suicide, (4) Difficulty with work and activities, (5) Psychic anxiety, (6) Somatic anxiety, and (7) General somatic symptoms. By explicitly employing this configuration, our research aims to identify optimal remission cut-off scores for the Thai elderly population and contribute to more tailored, evidence-based assessment standards in geriatric psychiatry.

However, practical limitations exist with more extended versions of the HAMD. Elderly patients may experience fatigue or cognitive limitations, and clinicians face time constraints, especially in busy outpatient clinics or research settings with high subject volumes. These realities have led to efforts to develop shorter, more focused versions of HAMD (12). The HAM-D7, which consists of only seven core items, was created to provide a quick yet valid assessment of the principal symptoms of major depression, focusing on domains such as mood, guilt, work and activities, psychomotor retardation, anxiety, and somatic symptoms. This abbreviated version can be administered in as little as 2–3 minutes by trained evaluators, making it highly practical in both clinical and research settings.

Despite its brevity, the HAM-D7 has demonstrated adequate validity and reliability for both initial assessment and monitoring of remission (13). Remission—defined as the near or complete resolution of depressive symptoms—is the goal of depression treatment and is associated with better functional and long-term outcomes. Remission status in patients with major depressive disorder can be effectively assessed using the 7-item Hamilton Depression Rating Scale (HAMD-7), which offers a concise yet reliable evaluation of core depressive symptoms. A HAMD-7 score at or below the established cut-off is generally regarded as indicative of remission. Traditionally, a score of ≤ 3 has been used to define complete remission, reflecting the absence or minimal presence of depressive symptoms (13). However, emerging evidence—including findings from elderly Thai populations—suggests that a cut-off score of ≤ 4 may be more appropriate to account for age-related somatic or subthreshold symptoms that are common in this demographic. Scores that fall between the remission threshold and the range that indicates active depression (typically 5–7) are interpreted as partial remission, denoting residual symptoms that do not meet full diagnostic criteria for an episode of major depression but may still impact functioning. Scores exceeding this range suggest a lack of remission and the continuing presence of clinically significant depressive symptoms. Thus, determining and utilizing appropriate HAMD-7 cut-offs is essential for accurately monitoring treatment response and guiding clinical decision-making, especially in elderly patients with depression.

Recently, further development in the field of depression assessment has produced the GRID-HAMD, a technically enhanced and standardized version of the original HAMD (14). The GRID system was introduced to improve the consistency, reliability, and transparency of depression rating by clearly defining each item’s assessment process. Unlike the conventional single-dimensional scoring, the GRID-HAMD uses a dual-axis framework that rates both the frequency and intensity of each symptom. Each component is scored separately and then combined to yield a final score for the item. The GRID approach provides more detailed guidance for raters, thereby reducing ambiguity and inter-rater variability—an essential advancement in both multicenter research and clinical practice. The GRID-HAM-D7 is the 7-item version adhering to this structured GRID methodology, with scores ranging up to a maximum of 26 points.

Usage of the HAM-D7 and GRID-HAM-D7 among elderly patients has increased (15), particularly as these populations are recognized to benefit from brief but reliable depression assessment (16). Although the popularity of brief depression rating scales is growing among elderly populations, most published studies, including Henrique-Araújo and colleagues (15), have evaluated the 17-item (GRID-HAMD-17) and 21-item (GRID-HAMD-21) versions, rather than the 7-item forms (HAM-D7 or GRID-HAM-D7) specifically considered in our study. However, most published remission cut-off scores are derived from cohorts of younger adults or mixed-age samples, and they may not translate directly to older individuals (17, 18). Aging is associated with neurobiological changes, medical comorbidities, and polypharmacy, all of which may influence the expression and assessment of depressive symptoms. In addition, the cultural context may further influence how symptoms are reported, detected, and understood, particularly in non-Western populations, such as those in Thailand. Reliable identification of remission is crucial for tailoring treatment plans, minimizing unnecessary medication exposure, and enhancing patient outcomes.

Despite the importance of accurate remission assessment among elderly Thais with depression, there are currently no established optimal cut-off scores for the GRID-HAM-D7 in this population. While the present study utilizes data collected between October 2012 and March 2015, the relationship between depressive symptom severity and diagnostic remission, as well as the rating tools themselves, has not fundamentally changed in the intervening years. Moreover, extensive, locally validated studies focused on the Thai elderly remain scarce, and the findings are immediately relevant for clinical assessment today. By evaluating and defining the optimal cut-off points for the GRID-HAM-D7 in this understudied population, our study addresses a persistent gap in geriatric depression care. Establishing a population-specific standard for remission can enhance the precision of clinical care, promote more effective monitoring in both research and practice, and ultimately improve the lives of older adults living with depression.

## Materials and methods

This research was conducted as part of the DAS Program, a comprehensive one-year observational and follow-up cohort study designed to investigate mental health conditions among the elderly in Thailand. The study recruited a total of 803 older adults who initially presented to psychiatric or geriatric outpatient clinics across four major tertiary care centers in the country. Data collection took place from October 2012 to March 2015. For the present analysis, only baseline data were utilized. The overarching goal of the DAS Program was to assess the presence of any psychiatric diagnosis in elderly individuals presenting with a range of chief complaints during their first encounter with the attending physician.

All study procedures were reviewed and approved by the Central Research Ethics Committee of Thailand, the Ethics Committee of Prasat Neurological Institute, and the Ethics Committee of Songkhla Rajanagarindra Psychiatric Hospital. Participation in the study was entirely voluntary, and all participants received a thorough explanation of the research objectives and procedures. Written informed consent was obtained from all subjects before participation.

### Participants

A total of 803 elderly Thai patients were consecutively recruited from four tertiary care centers across different regions of Thailand. Eligibility criteria for enrollment included: (1) age 60 years or older; (2) new patients who had not previously received services at the participating outpatient facilities; and (3) presenting with at least one self-reported symptom, such as feelings of boredom, sadness, sleep disturbances, loss of appetite, fatigue, poor memory, or unexplained physical complaints. Patients were excluded if they: (1) experienced severe physical symptoms that would interfere with the clinical interview; (2) had a diagnosis of substance use disorder; (3) exhibited significant language barriers impeding communication with the research team; (4) were diagnosed with dementia or other major cognitive impairments; or (5) had a history of schizophrenia, schizoaffective disorder, or manic episodes. This careful inclusion and exclusion process ensured that the study population was representative of elderly individuals seeking outpatient mental health services, while minimizing confounding factors that could interfere with the accurate assessment of depressive and psychiatric symptoms.

### Measurements

#### Thai version of HAM-D7 and GRID-HAM-D7

The Thai version of the HAM-D7 used in this study strictly follows the 7-item configuration validated by *McIntyre and colleagues* (13). The scale assesses the following seven depressive symptom domains: (1) Depressed mood, (2) Feelings of guilt, (3) Suicide, (4) Difficulty with work and activities, (5) Psychic anxiety, (6) Somatic anxiety, and (7) General somatic symptoms. This explicit listing ensures clarity in the specific version of the HAM-D7 evaluated and supports accurate interpretation and reproducibility of our results. The Thai version of the HAMD-7 was developed following rigorous guidelines for translation and cultural adaptation of psychological instruments (19). Permission was obtained from the original authors before translation. Initially, the first author translated the English HAMD-7 into Thai. Next, a bilingual expert, a native speaker of both English and Thai with professional experience in book writing, performed a back-translation from Thai to English. The original and back-translated versions were then compared for semantic equivalence. Discrepancies were discussed and resolved by the translation team, resulting in the finalized Thai version of the HAMD-7.

The internal consistency of the Thai HAMD-7 proved acceptable, with a Cronbach’s alpha of 0.72. When a cut-off score of 4 was applied to define remission, the scale demonstrated high specificity (97%). The instrument also exhibited excellent discrimination capacity, with an area under the curve (AUC) of 0.97 (95% CI 0.94–1.01) and a standard error of 0.019.

As part of the research, the GRID-HAMD system was subsequently adopted. The GRID-HAMD was developed to further enhance the usability of the scale by introducing a more structured format for rating items, thus improving clarity, rater consistency, and overall accuracy. The GRID methodology facilitated standardized ratings of both symptom frequency and intensity, making it suitable for use in multi-center studies and for ensuring reliable symptom assessment among elderly Thai participants.

#### Mini-International Neuropsychiatric Interview, version 5.0

All participants were diagnosed with the presence of depressive disorder according to DSM-IV by using the Mini-International Neuropsychiatric Interview (MINI), version 5.0. (20) MINI is a structured interview used for diagnosing psychiatric disorders according to DSM-IV. The Thai version of MINI version 5.0 has been widely used among psychiatrists and trained clinicians. The Kappa coefficients of MDD are 0.87. (21, 22).

#### HAM-D7 training

Proper assessment of depression with the HAM-D7 requires standardized rater training. To ensure inter-rater reliability for the Thai version of the HAM-D7, a pre-study training session was conducted for all participating physicians and clinicians. Although the clinicians were already familiar with the HAM-D17 in their routine clinical practice, the training specifically focused on the administration, scoring procedures, and interpretation of the Thai GRID-HAM-D7.

During the training, particular emphasis was placed on the unique aspects of the GRID-HAM-D7, including the structured approach to rating frequency and intensity for each item. Raters were guided step-by-step through the standardized questions and instructed to record responses according to the frequencies and symptom details reported by participants. The primary aim was to achieve consistency in rating rather than to assess the clinical expertise of each doctor.

Inter-rater reliability was rigorously assessed, and 100% agreement in scoring was achieved prior to study initiation. This process ensured that all evaluations throughout the study were performed consistently and in accordance with the GRID-HAM-D7’s structured methodology.

### Statistical analysis

Descriptive statistics, including frequencies and percentages, were used to summarize the demographic and clinical characteristics of the sample. The diagnostic performance of the Thai version of the GRID-HAM-D7 was evaluated by calculating its sensitivity and specificity, using the Mini International Neuropsychiatric Interview (MINI) based on DSM-IV criteria as the gold standard for depression diagnosis. In addition, the positive predictive value (PPV) and negative predictive value (NPV) were determined to assess the likelihood that a given GRID-HAM-D7 score accurately identifies remission and non-remission, respectively.

To identify the optimal cut-off score for remission on the GRID-HAM-D7, the Youden Index (J) was calculated, which maximizes the sum of sensitivity and specificity, indicating the threshold with the best overall diagnostic accuracy. Furthermore, Receiver Operating Characteristic (ROC) curve analysis was conducted, and the area under the curve (AUC) was computed to assess the discriminative ability of the GRID-HAM-D7 in distinguishing between remitted and non-remitted cases.

All statistical analyses were performed using the Statistical Package for the Social Sciences (SPSS) version 22.0 for Windows (IBM Corporation, Armonk, NY, USA) and MedCalc version 12.3 (MedCalc Software, Mariakerke, Belgium).

## Results

**Table 1** presents the demographic and clinical characteristics of the study participants. The majority of the participants were female and married. More than half were unemployed and reported low-income levels. Among the 803 participants, 23.67% were diagnosed with a depressive disorder, and of these, 72.6% had major depressive disorder (MDD), which corresponds to 17.19% of the total sample (as shown in **Table 2**). When dysthymic disorder was excluded from the analysis, the prevalence of MDD (including cases of double depression) was 138 out of 760 participants, yielding a rate of 18.16%.

**Table 1:** Demographic data (N = 803).

Variables	Values
Gender	
Male, n (%)	239 (30)
Female, n (%)	557 (70)
Mean age, year, SD (min-max)	69.24, 6.88 (60-89)
Mean education, year, SD (min-max)	6.63, 4.9 (0-21)
Marital status, n (%)	
Single	29 (3.61)
Married	509 (63.39)
Divorce	36 (4.48)
Widow	228 (28.39)
Employment	
Employed	353 (45.1)
Unemployed	429 (54.9)
Income (THB/Month)	
<5000	469 (59.4)
5001-10,000	110 (13.9)
10,001-20,000	114 (14.4)
>20,000	96 (12.2)

**Table 2:** Number and percentage of depressive disorders (n=190).

Depression types	Number (n)	Percentage (%)
Major depressive disorder	138	17.19
Dysthymia	40	4.98
Double Depression	12	1.49
Total	190	23.66

When comparing clinical subgroups, the mixed anxiety and depressive disorder group had the highest mean HAMD-7 scores, indicating greater symptom severity within this subgroup compared to those with other depressive diagnoses (**Table 3**).

**Table 3:** Comparison of the HAMD scores of participants without and with depressive disorders, anxiety disorders, and both.

Disorder	Mean	SD	*F* (3,795)	P-value
None of any	2.04	2.327	261.95	<0.001
Depressive disorder	9.49	6.151		
Anxiety disorder	6.10	3.780		
Mixed anxiety and depressive disorder	13.75	6.044		

HAMD, Hamilton Rating Scale for Depression.

**Figure 1:**
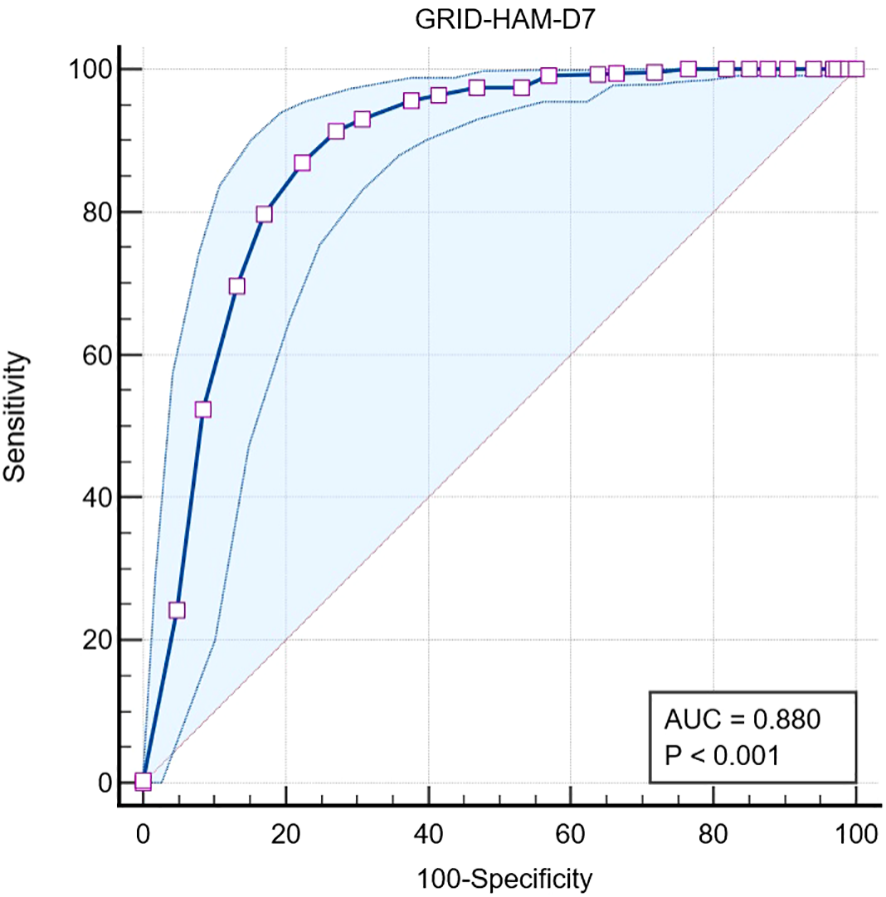
The ROC curve and area under the curve (AUC) for GRID-HAM-D7 in identifying remission in any depressive disorder.

**Figure 1** shows the area under the ROC curve (AUC) was 0.88, standard error of 0.017, 95% Confidence interval of 0.86 to 0.90, z statistic 22.56, and p = <0.0001.

**Table 4** presents the diagnostic performance of various GRID-HAM-D7 cut-off scores in identifying remission among elderly patients. The optimal threshold was found at a cut-off score of ≤ 4, which yielded the highest Youden index (J = 0.64), indicating the best balance between sensitivity and specificity for this population. At this criterion, the sensitivity was 88.86%, demonstrating the tool’s strong capability to accurately detect patients who have achieved remission. The specificity was 77.66%, reflecting a similarly high accuracy rate for identifying those who have not reached remission. Furthermore, the positive predictive value (PPV) was 92.6%, indicating that the vast majority of individuals classified as remitted truly were in remission. The negative predictive value (NPV) was 64.6%, suggesting that over half of the non-remission classifications were correct, though with some potential for false negatives. Additionally, the positive likelihood ratio (+LR) was 3.89, which substantially increases the likelihood of remission with a positive test result, while the negative likelihood ratio (−LR) was 0.17, indicating that a negative result significantly reduces the probability of remission. Together, these findings reinforce that a cut-off score of ≤ 4 on the GRID-HAM-D7 is both sensitive and specific for assessing remission from any depressive disorder in the elderly Thai population.

**Table 4:** GRID-HAM-D7 cut-off scores and coordinates of the ROC curve for predicting remission in any depressive disorders.

Criterion	Sensitivity	95% CI	Specificity	95% CI	+LR	95% CI	-LR	95% CI	PPV	95% CI	NPV	95% CI
≤1	52.38	48.3 - 56.4	91.49	86.5 - 95.1	6.15	3.83 - 9.89	0.52	0.47 - 0.57	95.2	92.5 - 97.0	37.2	35.1 - 39.5
≤2	69.62	65.8 - 73.3	86.7	81.0 - 91.2	5.24	3.62 - 7.57	0.35	0.31 - 0.40	94.4	92.1 - 96.1	46.8	43.6 - 50.2
≤3	79.8	76.4 - 82.9	82.98	76.8 - 88.1	4.69	3.41 - 6.44	0.24	0.21 - 0.29	93.8	91.7 - 95.4	55.9	51.7 - 60.1
≤4*	86.86	83.9 - 89.4	77.66	71.0 - 83.4	3.89	2.97 - 5.08	0.17	0.14 - 0.21	92.6	90.6 - 94.3	64.6	59.5 - 69.4
≤5	91.3	88.8 - 93.4	72.87	65.9 - 79.1	3.37	2.66 - 4.26	0.12	0.091 - 0.16	91.6	89.6 - 93.2	72.1	66.3 - 77.2
≤6	93.1	90.8 - 95.0	69.15	62.0 - 75.7	3.02	2.43 - 3.74	0.1	0.073 - 0.14	90.7	88.7 - 92.4	75.6	69.5 - 80.8
≤7	95.57	93.6 - 97.1	62.23	54.9 - 69.2	2.53	2.10 - 3.04	0.071	0.048 - 0.10	89.1	87.2 - 90.8	81.3	74.7 - 86.4
≤8	96.39	94.6 - 97.7	58.51	51.1 - 65.6	2.32	1.96 - 2.75	0.062	0.040 - 0.095	88.3	86.4 - 89.9	83.3	76.5 - 88.5
≤9	97.54	96.0 - 98.6	53.19	45.8 - 60.5	2.08	1.79 - 2.43	0.046	0.028 - 0.078	87.1	85.3 - 88.7	87	79.9 - 91.8
≤10	97.54	96.0 - 98.6	46.81	39.5 - 54.2	1.83	1.60 - 2.10	0.053	0.031 - 0.089	85.6	83.8 - 87.2	85.4	77.7 - 90.8

**Figure 2:**
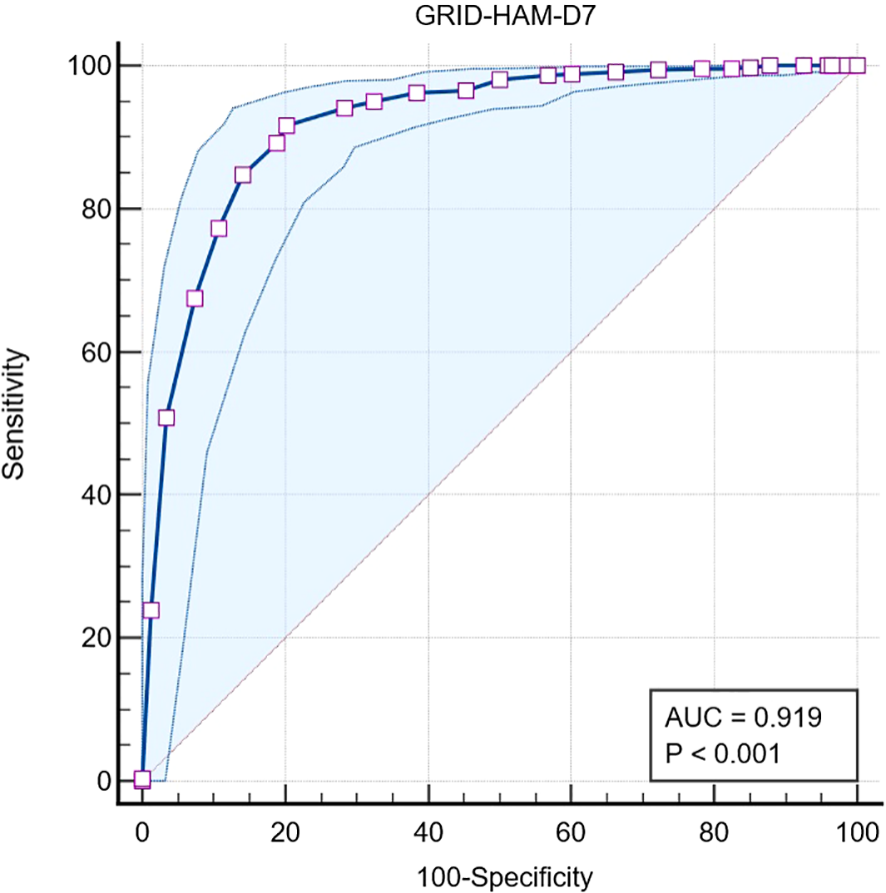
The ROC curve and area under the curve (AUC) for GRID-HAM-D7 in identifying remission in major depressive disorder.

**Figure 2** shows the area under the ROC curve (AUC) was 0.92, standard error of 0.02, 95% Confidence interval of 0.90 to 0.94, z statistic 27.69, and p = <0.0001.

**Table 5** presents the diagnostic performance of various GRID-HAM-D7 cut-off scores in identifying remission among elderly patients. The optimal threshold was found at a cut-off score of ≤ 6, which yielded the highest Youden index (J = 0.71), indicating the best balance between sensitivity and specificity for this population. At this criterion, the sensitivity was 91.68%, demonstrating the tool’s strong ability to accurately detect patients who have achieved remission. The specificity was 79.73%, reflecting a similarly high accuracy rate for identifying those who have not reached remission. Furthermore, the positive predictive value (PPV) was 95.2%, indicating that the vast majority of individuals classified as remitted truly were in remission. The negative predictive value (NPV) was 68.6%, indicating that over half of the non-remission classifications were accurate, although with some potential for false negatives. Additionally, the positive likelihood ratio (+LR) was 4.52, which substantially increases the likelihood of remission with a positive test result, while the negative likelihood ratio (−LR) was 0.10, indicating that a negative result significantly reduces the probability of remission. Together, these findings reinforce that a cut-off score of ≤ 6 on the GRID-HAM-D7 is both sensitive and specific for assessing remission from major depressive disorder in the elderly Thai population.

**Table 5:** GRID-HAM-D7 cut-off scores and coordinates of the ROC curve for predicting remission in major depressive disorder (MDD).

Criterion	Sensitivity	95% CI	Specificity	95% CI	+LR	95% CI	-LR	95% CI	PPV	95% CI	NPV	95% CI
≤1	50.85	46.9 - 54.8	96.62	92.3 - 98.9	15.05	6.34 - 35.74	0.51	0.47 - 0.55	98.5	96.5 - 99.4	31	29.2 - 32.8
≤2	67.49	63.7 - 71.1	92.57	87.1 - 96.2	9.08	5.13 - 16.07	0.35	0.31 - 0.40	97.6	95.7 - 98.6	39.4	36.5 - 42.3
≤3	77.35	73.9 - 80.5	89.19	83.0 - 93.7	7.15	4.50 - 11.39	0.25	0.22 - 0.30	96.9	95.2 - 98.0	47.3	43.5 - 51.1
≤4	84.75	81.7 - 87.4	85.81	79.1 - 91.0	5.97	4.01 - 8.89	0.18	0.15 - 0.22	96.3	94.6 - 97.5	56.2	51.4 - 60.9
≤5	89.21	86.6 - 91.5	81.08	73.8 - 87.0	4.72	3.37 - 6.59	0.13	0.11 - 0.17	95.4	93.7 - 96.7	63.2	57.6 - 68.4
≤6*	91.68	89.3 - 93.7	79.73	72.3 - 85.9	4.52	3.28 - 6.23	0.10	0.080 - 0.14	95.2	93.5 - 96.5	68.6	62.6 - 74.1
≤7	94.14	92.1 - 95.8	71.62	63.6 - 78.7	3.32	2.57 - 4.29	0.082	0.059 - 0.11	93.6	91.8 - 95.0	73.6	66.8 - 79.4
≤8	95.07	93.1 - 96.6	67.57	59.4 - 75.0	2.93	2.32 - 3.70	0.073	0.051 - 0.10	92.8	91.1 - 94.2	75.8	68.6 - 81.7
≤9	96.3	94.5 - 97.6	61.49	53.1 - 69.4	2.5	2.04 - 3.07	0.06	0.040 - 0.091	91.6	89.9 - 93.1	79.1	71.5 - 85.1
≤10	96.61	94.9 - 97.9	54.73	46.3 - 62.9	2.13	1.79 - 2.55	0.062	0.040 - 0.096	90.3	88.7 - 91.8	78.6	70.4 - 85.1

## Discussion

This study provides novel, evidence-based recommendations for the use of the GRID-HAM-D7 in identifying remission in elderly Thai patients with depressive disorders, drawing important distinctions between any depressive disorder and major depressive disorder (MDD). Our findings demonstrate that a cut-off score of ≤ 4 is most appropriate for identifying remission in any depressive disorder, while a higher threshold of ≤ 6 is optimal for MDD. These results highlight an essential nuance in the diagnostic assessment of depression among older adults. This group remains underrepresented in much of the global research on depression rating scale validation.

The differentiation between optimal cut-off values for any depressive disorder and MDD reinforces the growing understanding that late-life depression is highly heterogeneous, both in presentation and severity (23, 24). Elderly patients often report more somatic symptoms, greater cognitive complaints, and less prominent affective symptoms compared to younger cohorts (25–27). Recent work suggests that standard HAM-D or GRID-HAM-D7 thresholds established in younger, predominantly Western samples may inadequately capture remission in geriatric or East Asian populations (28). Our findings align with this perspective, showing that traditional cut-offs (≤ 3) may be overly restrictive, potentially failing to identify significant numbers of elderly patients who have, in fact, achieved a clinically meaningful remission.

The diagnostic accuracy parameters observed in our study support the clinical utility of the GRID-HAM-D7 where a higher cut-off for MDD (≤ 6) yielded a Youden index of 0.71, with improved sensitivity (91.68%) and specificity (79.73%) over the ≤ 4 cut-off for any depressive disorder (Youden index 0.64; sensitivity 88.86%, specificity 77.66%). This echoes studies by Zimmerman and colleagues (29), who caution that overly stringent remission thresholds may not be appropriate for populations with more chronic or somatic symptom profiles, such as older adults. The improved positive predictive value (PPV) in our sample suggests that clinicians can be confident when GRID-HAM-D7 scores indicate remission, especially in the context of MDD, where nearly all individuals classified as remitted indeed met clinical criteria for remission.

Our results also emphasize the importance of culturally and contextually appropriate benchmarks. Cultural factors—including differing attitudes toward mental health, somatic expression of psychological distress, and stigma—directly influence both the reporting and clinical recognition of depressive symptoms (30–32). In the Thai context, mental health stigma may drive underreporting of emotional symptoms and a preference for expressing distress through physical complaints. This underscores the value of the GRID-HAM-D7, which offers a dimensional approach that better captures the breadth of presentation characteristic of late-life depression in non-Western settings (33).

Further, our results are based on prior psychometric evaluations of the GRID-HAM-D17. Williams and colleagues (14) have demonstrated the advantages of the GRID format in enhancing inter-rater reliability and symptom assessment granularity. In elderly samples, these strengths may be particularly relevant, as cognitive impairment or medical comorbidity further complicate clinical ratings and subjective self-reporting (34). By elucidating which cut-off scores maximize diagnostic accuracy in the elderly, our findings enhance the utility of the GRID-HAM-D7 as both a research and a clinical tool.

However, the moderate negative predictive values (NPV) in both categories indicate that caution should be exercised in interpreting negative test results; a substantial fraction of non-remission classifications may still represent remitted cases, echoing the complexity of depression assessment among the elderly as highlighted in previous research (35, 36). This finding suggests that, particularly in “borderline” cases, comprehensive clinical evaluation—including collateral information from caregivers and consideration of the broader psychosocial context—remains essential.

Finally, our results support calls for future research to move beyond static cut-off validation, toward longitudinal studies that examine how remission, as assessed by these thresholds, correlates with functional outcomes, relapse rates, and patient quality of life over time (37, 38). Such data are vital for translating diagnostic accuracy into meaningful improvement in patient care.

It is important to note that our data were collected between 2012 and 2015. Nevertheless, the clinical and psychometric landscape of late-life depression—and the diagnostic relevance of the GRID-HAM-D7—has remained fundamentally unchanged since. Large-sample, population-specific studies in elderly Thai populations are still sparse, and thus our findings remain highly pertinent for contemporary clinical care and research. Our work helps bridge the enduring gap in evidence-based practice for this growing demographic.

### Limitations

Several limitations warrant consideration. First, although data collection occurred between 2012 and 2015, there remains a lack of recent large-scale studies focusing on remission cut-offs for the GRID-HAM-D7 in elderly Asian or Thai populations. Recent literature continues to emphasize the scarcity of population-specific validation studies for depression assessment tools in older adults, which underscores the ongoing relevance and contribution of our findings despite the dated dataset. Second, the sample was drawn from select clinical settings, which may constrain the generalizability of the results to broader, community-based elderly populations within Thailand and beyond. Future research should broaden its scope to encompass more diverse patient demographics and multiple care settings. Third, the assessments relied entirely on the clinician-rated GRID-HAM-D7. While this instrument is validated and robust, complementing it with patient self-report scales could provide additional perspectives, particularly on subjective well-being and symptomatology that clinicians may miss. Fourth, although this study identified optimal cut-offs corresponding to strong diagnostic accuracy, we did not directly link these thresholds to longitudinal outcomes or treatment trajectories. Prospective studies are recommended to examine whether remission, as classified by these cut-offs, translates into meaningful improvements in daily functioning, relapse rates, or overall well-being. Fifth, cultural factors, including stigma and the somatization of psychological distress, may have influenced symptom reporting. Qualitative research may clarify how cultural context affects both the diagnosis and self-perception of remission, guiding refinement of assessment tools and clinical interviewing in Thailand and similar settings. Lastly, although participants with severe physical illnesses or cognitive impairments were excluded to minimize major confounders, there remains substantial overlap between depressive symptoms, such as fatigue, somatic complaints, and cognitive slowing, and normal aging or comorbid medical conditions in elderly individuals. The potential for misattribution of symptoms between depression and age-related changes cannot be fully eliminated and should be considered when interpreting the findings.

## Conclusion

This study offers critical evidence for the use of the GRID-HAM-D7 in elderly Thai patients by establishing distinct, diagnosis-specific cut-off scores for remission—≤ 4 for any depressive disorder and ≤ 6 for MDD. These findings affirm the need for tailored assessment strategies that account for the heterogeneous presentation of depressive symptoms in later life and across diagnostic categories.

By providing empirically derived, population-specific cut-offs, this research supports more accurate identification of remission, informing clinical decision-making and potentially facilitating improved patient outcomes. Future research should validate these cut-offs in broader settings and link them to long-term recovery and quality of life, ensuring that older adults receive optimized, culturally sensitive care for depression as they age.

## Data Availability

The raw data supporting the conclusions of this article will be made available by the authors, without undue reservation.
